# Source-Specific Accumulation, Translocation, and Health Risks of Potentially Toxic Elements in Paddy Fields from Different Anthropogenic Impact Zones in Hunan Province, China

**DOI:** 10.3390/plants15121818

**Published:** 2026-06-12

**Authors:** Ying Huang, Pengyue Yu, Ruimin Chang, Zhiyan Xie, Zhi Huang, Jianwei Peng, Yaocheng Deng, Zhaojun Li

**Affiliations:** 1 State Key Laboratory of Efficient Utilization of Arid and Semi-Arid Arable Land in Northern China, Institute of Agricultural Resources and Regional Planning, Chinese Academy of Agricultural Sciences, Beijing 100081, China; 2 National Engineering Laboratory of High Efficient Use on Soil and Fertilizer, College of Resources, Hunan Agricultural University, Changsha 410128, China; yupengyue@stu.hunau.edu.cn (P.Y.); chang_ruimin@stu.hunau.edu.cn (R.C.); xiezhiyan@stu.hunau.edu.cn (Z.X.); huangzhi18714838265@stu.hunau.edu.cn (Z.H.); jianweipenglab@hunau.edu.cn (J.P.); 3 College of Environment & Ecology, Hunan Agricultural University, Changsha 410128, China; dengyaocheng@hunau.edu.cn

**Keywords:** rice, atmospheric deposition, potentially toxic elements, health risk assessment, source analysis

## Abstract

Potentially toxic element (PTE) contamination in rice poses significant food safety risks, particularly in regions with intensive agriculture, industry, and traffic. This study provides a systematic assessment of the accumulation, translocation, sources, and health risks of PTEs (As, Cd, Cr, Cu, Ni, Pb, Zn) in the atmospheric deposition–soil–rice system across four distinct anthropogenic source areas (industrial, peri-urban, rural, and roadside areas) in Hunan Province, China. The rural area was categorized as clean. Industrial areas had the highest soil pollution index, while roadside areas recorded the highest atmospheric deposition flux of Pb (19.95 μg/m^2^/day) and As (1.93 μg/m^2^/day). Correspondingly, industrial areas exhibited the highest Cd (0.38 mg/kg) and Pb (0.94 mg/kg) in rice grains, whereas roadside areas showed the highest Pb (1.40 mg/kg) and As (2.99 mg/kg) in leaves. The findings indicated that rice in roadside areas primarily accumulate PTEs through foliar absorption of atmospheric deposition, whereas in industrial and peri-urban areas it was primarily through root uptake and translocation of PTEs to rice grains, particularly for Cd and Pb. Source apportionment identified natural, industrial, and traffic as the three primary sources. The Bayesian mixing model revealed that the natural source contributed the highest proportion to rice grains (48.3–70.6%) across all four source areas. Except for natural sources, industrial sources dominated in industrial areas (29.1%), traffic emissions prevailed in roadside areas (19.4%), while mixed sources had the highest proportion in peri-urban areas (28.4%). Health risk assessment revealed that the total hazard index followed the order of peri-urban > industrial > roadside > rural areas, with rice ingestion being the dominant exposure pathway, accounting for over 90% of the total risk. The primary contributors to health risks were identified as As, Cd, and Pb, particularly in industrial and peri-urban areas. These findings provide a scientific basis for developing region-specific mitigation strategies tailored to the dominant contamination pathways in each area.

## 1. Introduction

Rice, a major global staple food crop, faces a severe challenge of potentially toxic element (PTE) contamination, which poses significant food safety and public health risks [1,2]. This problem is particularly serious in China, where rapid industrialization and urbanization have led to widespread PTE contamination of agricultural soils and atmospheric deposition [3,4]. Hunan Province is known as the “land of fish and rice”. It ranks first in China for both rice planting area and total production. In 2024, the province produced 30.78 million tons of grain [5]. At the same time, Hunan is called the “home of non-ferrous metals” due to its abundant mineral resources, including the world’s largest antimony reserves [6]. Moreover, there are extensive transportation networks, with numerous highways, railways, and urban roads connecting Hunan. The rapid growth in vehicle numbers has led to increased traffic emissions, which have become an important source of PTEs such as lead (Pb) and metalloid arsenic (As) in the environment [7]. This combination of intensive agriculture, heavy traffic, and extensive industrial activity has created complex PTE pollution. Industrial emissions, traffic exhaust, and agricultural practices all contribute to elevated metal levels in paddy soils and the atmosphere [8,9]. As a result, PTE accumulation in rice has become a major threat to regional food security and public health.

Previous studies have identified two main pathways for PTE accumulation in rice, which are root uptake from soil and foliar uptake from atmospheric deposition. The soil–root pathway is controlled by soil properties and metal bioavailability, and its mechanisms have been well studied [10,11]. The foliar uptake occurs when atmospheric deposition deposits PTEs onto leaf surfaces, where they can be absorbed and subsequently translocated to grains [12]. Recent studies have shown that PTEs deposited on leaves, especially those on fine particles, can be absorbed and moved to grains. In some cases, this pathway may contribute more to grain cadmium than root uptake [13,14]. The characteristics of PTEs in deposition and soil depend heavily on natural and anthropogenic sources. Industrial emissions, traffic exhaust, and urban dust have distinct compositions and produce distinct pollution patterns [15,16,17]. However, most current studies are limited in this scope. They often focus on one environmental medium, such as soil or air. They also tend to attribute pollution to a single dominant source. Few studies have systematically compared different source areas at a regional scale. In particular, there is little research tracing how PTEs move from deposition and soil through different organs to rice grains, and how this varies across areas with different emission sources.

The study selected six cities in Hunan Province: Changsha, Zhuzhou, Xiangtan, Liuyang, Liling, and Ningxiang, which represent the main areas of urbanization, industry, and agriculture in the province. We collected matched samples of atmospheric deposition, soil, and rice samples. The seven elements (As, Cd, Cr, Cu, Ni, Pb, Zn) were selected because they are all regulated under China’s Soil Environmental Quality-Risk Control Standard for Soil Contamination of Agricultural Land (GB15618-2018) [18] and are routinely included in risk assessments of agricultural soils and crops [4]. The PTE concentrations were analyzed, as well as assessed health risks and source apportionment. Our goals were to: (1) compare atmospheric deposition and soil pollution levels across different source areas; (2) explore PTE accumulation in different rice organs and identify the main pathways to grains; and (3) evaluate health risks for residents in each source area.

## 2. Results

### 2.1. Characteristics of PTE Accumulation in Atmospheric Deposition and Soil in Four Types of Anthropogenic Source Areas

**Figure 1:**
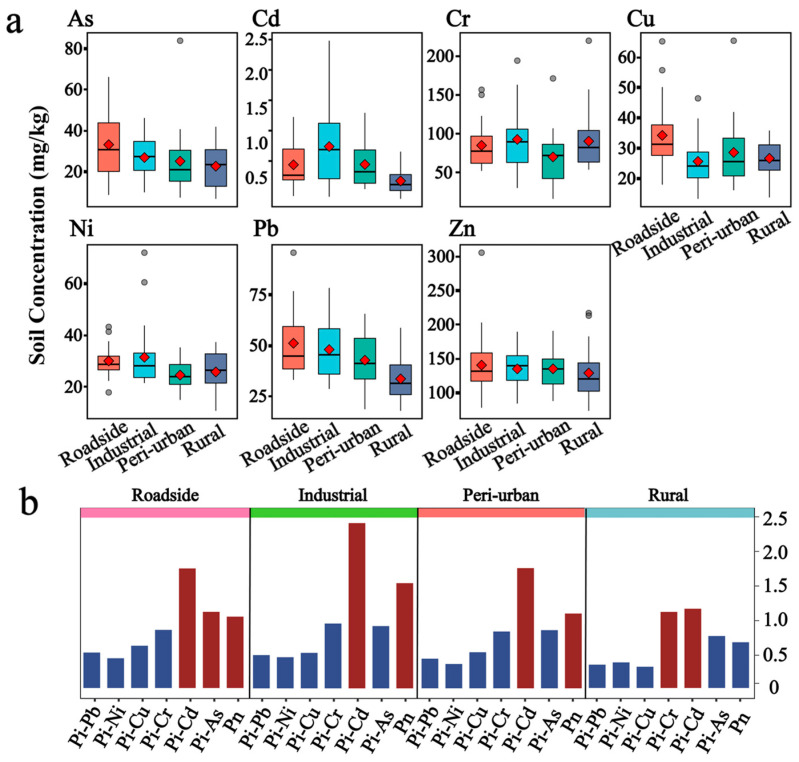
The PTE contents in soils (mg/kg) in different source areas (**a**); Nemerow Pollution Index in soils from different source areas (**b**). Pn represents the comprehensive pollution index for that area. Nemerow Index of different elements in soils in different functional areas (pollution index ≤ 0.7 clean; 0.7 < pollution index ≤ 1 fairly clean; 1 < pollution index ≤ 2 light contaminated; 2 < pollution index ≤ 3 moderate contamination; pollution index > 3 heavy pollution). Values greater than 1 are shown in red. The standard value uses China’s “Soil Environmental Quality-Risk Control Standards for Agricultural Soil Contamination” (5.5 < pH ≤ 6.5, GB15618-2018). The darker dots in the plots represent outliers, data points beyond 1.5 × IQR. The red square represents the average.

**Figure 2:**
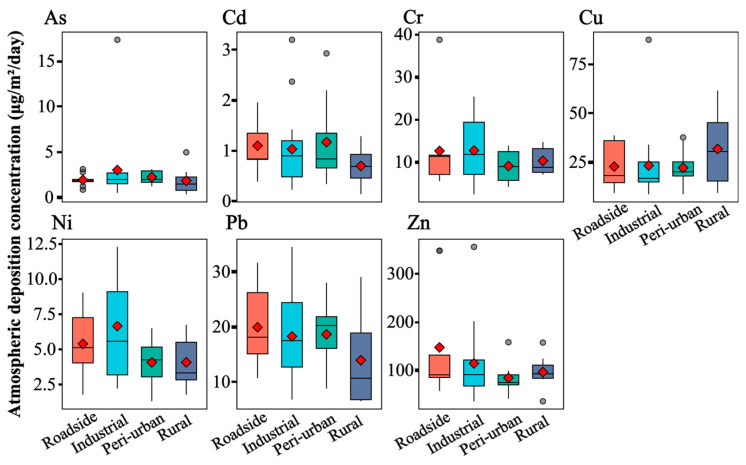
The PTE contents in atmospheric depositions (μg/m^2^/day) in different source areas. The darker dots in the plots represent outliers, data points beyond 1.5 × IQR. The red square represents the average.

The concentrations of PTEs in soil and atmospheric deposition samples collected from the study region exhibited significant spatial variation across the four source areas (Figure 1 and Figure 2). The rural areas generally showed the lowest PTE concentrations, with the mean As, Cd, Cu, Ni, Pb and Zn concentrations being 22.65 ± 10.34 mg/kg, 0.46 ± 0.21 mg/kg, 26.67 ± 6.02 mg/kg, 25.81 ± 7.86 mg/kg, 33.59 ± 11.01 mg/kg, and 128.91 ± 41.29 mg/kg, respectively, lower than in other areas. In contrast, in soil, the industrial areas exhibited the highest mean concentrations of As, Cd, Ni, and Pb, at 26.87 ± 10.48 mg/kg, 0.95 ± 0.55 mg/kg, 31.49 ± 12.76 mg/kg, and 48.05 ± 13.78 mg/kg, respectively (Figure 1a). The soil in roadside areas showed elevated levels of As and Pb, with mean concentrations of 33.14 ± 15.16 mg/kg and 51.17 ± 16.54 mg/kg. At the same time, Cu was most enriched in the peri-urban areas, reaching a mean of 28.62 ± 11.58 mg/kg. Figure 1a further illustrates the data distributions. For Cd, the rural area showed the narrowest range (0.20–0.88 mg/kg) and lowest median (0.41 mg/kg), while the industrial area had the widest range (0.23–2.48 mg/kg). For Pb, the rural area had the lowest median (31.39 mg/kg) and a narrow range, while the roadside area exhibited the highest maximum (95.85 mg/kg). There were significant differences in soil among the four areas for As (*p* < 0.001), Cd (*p* < 0.001), Cr (*p* = 0.01), Cu (*p* < 0.001), Ni (*p* < 0.001), and Pb (*p* < 0.001), while Zn showed no significant difference (*p* = 0.33) (Table S4). These results confirm stronger anthropogenic impact in industrial and roadside areas compared with the rural reference.

Atmospheric deposition also displayed distinct spatial patterns (Figure 2). The rural area generally had the lowest atmospheric deposition fluxes for most elements; Cd and Pb averaged 0.70 ± 0.42 μg/m^2^/day and 13.95 ± 9.63 μg/m^2^/day, substantially lower than in other areas. In contrast, the roadside area recorded the highest mean deposition fluxes for As, Cd, Ni, and Pb, at 1.93 ± 0.69 μg/m^2^/day, 1.10 ± 0.55 μg/m^2^/day, 5.40 ± 2.30 μg/m^2^/day, and 19.95 ± 6.94 μg/m^2^/day, respectively. The industrial area showed elevated Cd and Pb deposition, with mean fluxes of 1.03 ± 0.81 μg/m^2^/day and 18.29 ± 8.16 μg/m^2^/day. The peri-urban area exhibited moderate deposition levels, with Cd and Pb fluxes of 1.17 ± 0.85 μg/m^2^/day and 18.66 ± 6.15 μg/m^2^/day, respectively. Moreover, deposition fluxes in the peri-urban area fell between those in the industrial and roadside areas, reflecting the combined influence of industrial, traffic, and residential sources. Notably, the flux of Cu in atmospheric deposition was highest in the rural area at 31.82 ± 19.66 μg/m^2^/day. The atmospheric deposition fluxes of Cd (*p* < 0.05), Ni (*p* < 0.001), Pb (*p* < 0.01), Cr (*p* < 0.05), Cu (*p* < 0.05), and Zn (*p* < 0.001) differed significantly among the four areas, while As was non-significant (*p* = 0.053) (Table S4).

### 2.2. Distribution of PTEs in Rice Plants in Four Anthropogenic Source Areas

**Figure 3:**
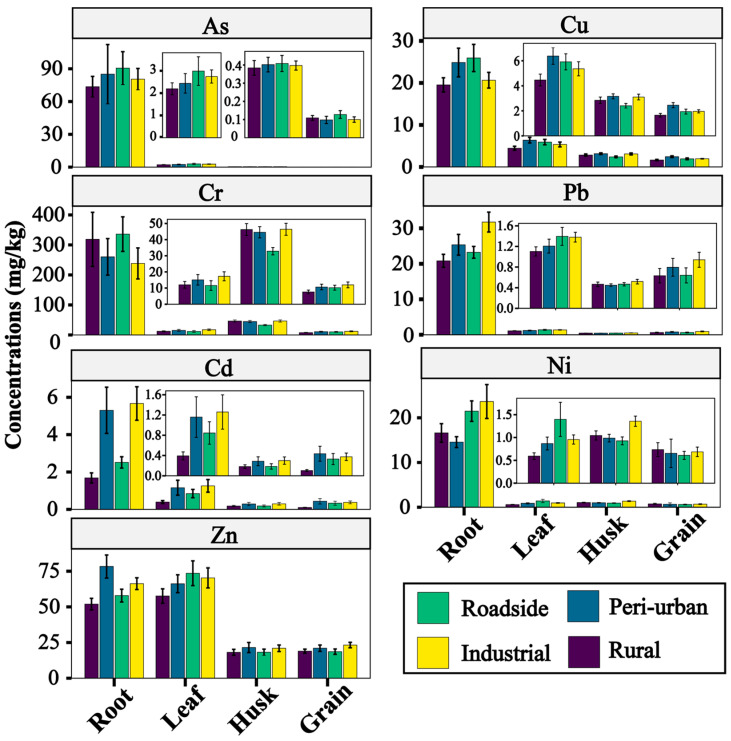
The PTE concentrations in different parts of the rice plant (mg/kg).

The concentrations of PTEs in different rice tissues exhibited pronounced spatial variation across the four source areas (Figure 3). For roots, Cd, Cu, Ni, Pb and Zn showed significant differences among the four areas (*p* < 0.01), while As and Cr did not (*p* > 0.05) (Table S4). The rural area consistently recorded the lowest PTE concentrations in roots, leaves, and rice grain, with a mean concentration of Cd in rice grain (0.10 ± 0.08 mg/kg), which was below the standard. In contrast, in the industrial area, the highest accumulation was shown in roots with mean concentrations as Cd (5.67 ± 4.29 mg/kg), Pb (31.75 ± 13.42 mg/kg), Ni (23.64 ± 18.20 mg/kg), and Cr (238.28 ± 247.74 mg/kg). Moreover, rice grain also exhibited higher levels of Cd (0.38 ± 0.35 mg/kg) and Pb (0.94 ± 0.70 mg/kg), exceeding the Chinese food safety standard (GB 2762-2022) [19], which sets maximum permissible levels of 0.20 mg/kg for both Cd and Pb in rice grain. For leaves, Cd, Cr, Cu, Ni, Pb and Zn differed significantly (*p* < 0.05), except As (Table S4). In the roadside area, leaves contained the highest concentration of Pb (1.40 ± 0.89 mg/kg) and As (2.99 ± 3.26 mg/kg) among all regions. The concentration of Pb in the root in the roadside area (23.23 ± 8.45 mg/kg) was also substantial but lower than in the industrial area. For rice grains, Cd, Cr, Cu, Pb and Zn exhibited significant differences (*p* < 0.05) (Table S4). Rice grains in the roadside area exhibited relatively high levels of As (0.13 ± 0.11 mg/kg) and Pb (0.64 ± 0.73 mg/kg), while those of Zn (18.61 ± 9.57 mg/kg) and Cu (1.94 ± 1.01 mg/kg) were at moderate levels. In the peri-urban area, rice grains exhibited the highest concentration of Cd (0.44 ± 0.66 mg/kg) among all regions. The levels of Pb and As in the rice grains were relatively high, at 0.80 ± 0.77 mg/kg and 0.10 ± 0.07 mg/kg, respectively. The concentration of Cd in root (5.30 ± 5.53 mg/kg) was as high as in industrial areas.

### 2.3. Bioconcentration and Transfer of PTEs in Rice Tissues Across Four Source Areas

By calculating the bioconcentration factor (*BCF*) and transfer factor (*TF*) for each element, significant differences were observed in the uptake and internal translocation of PTEs in rice tissues (Table 1). Among all areas, Cd exhibited the highest *BCF_soil-to-root_*, ranging from 4.24 in the roadside area to 6.09 in the peri-urban area, indicating that rice roots have a strong capacity to accumulate Cd. Meanwhile, the *BCF_atmosphere-to-leaf_* for Cd reached 6.61 in the roadside area, substantially higher than in other regions. The index of *TF_Root-to-leaf_* for all elements except Zn were consistently below 1 across all regions, demonstrating that roots effectively sequester most PTEs and restrict upward transport. Additionally, *TF_leaf-to-grain_* for Pb exceeded 1 in all areas (1.14–1.84), confirming efficient foliar-to-grain translocation. The *TF_root-to-grain_* for Cd ranged from 0.07 to 0.13, indicating that relatively few of the PTEs fixed by the roots are directly transported to the grains. The *TF_leaf-to-husk_* was notably high for Ni (1.44–2.70) and Cr (8.53 in the peri-urban area), indicating preferential translocation of these elements to the husk. Notably, *TF_husk-to-grain_* for Pb exceeded 1 in all regions, reaching 3.16 in the industrial area, confirming that Pb accumulated in husk is easily mobilized into the rice grains. Cd also showed elevated *TF_husk-to-grain_* (1.15–2.00).

### 2.4. Source Apportionment and Validation of PTE Accumulation in Soil and Rice Grains

**Figure 4:**
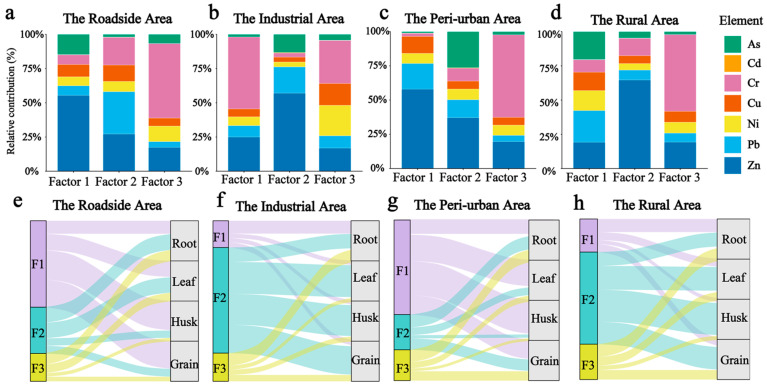
Elemental fingerprints in environmental samples from roadside (**a**), industrial (**b**), peri-urban (**c**), and rural (**d**) areas, as analyzed using PMF model. Each bar represents the relative contribution (%) of each element within a factor. Source contributions to different rice organs (root, leaf, husk, and grain) estimated by the Bayesian mixing model (MixSIAR) for the roadside (**e**), industrial (**f**), peri-urban (**g**), and rural (**h**) areas.

The Positive Matrix Factorization (PMF) analysis was used to identify potential sources of PTEs and quantify their contributions, yielding three factors per area with distinct elemental fingerprints (Figure 4a–d). In the roadside area, Factor 1 was dominated by Zn (55.4%), As (14.7%), and showed relatively even distribution of other elements, indicating a natural source. Factor 2 was characterized by high Pb (30.9%) and Cr (20.2%), indicating traffic emissions, vehicle exhaust, and brake wear. Factor 3 was dominated by Cr (54.4%) and Ni (11.3%), pointing to industrial sources, electroplating, and alloy manufacturing. In the industrial area, Factor 1 was dominated by Cr (52.5%) and Zn (25.0%), indicating industrial sources. Factor 2 showed a relatively even distribution of elements, indicating a natural source. Factor 3 was characterized by high loadings of Cr (31.4%) and Ni (22.3%), indicative of industrial sources such as electroplating or alloy manufacturing. Factor 1 in the peri-urban area and Factor 2 in the rural area exhibited similar loading patterns to Factor 1 in the roadside area and Factor 2 in the industrial area, suggesting a natural source. Factor 2 in the peri-urban area was dominated by As (26.3%), Pb (13.1%), Cr (9.4%), Ni (7.8%), and Zn (37.2%), clearly indicating mixed pollution sources. Factor 3 in the peri-urban area was dominated by Cr (60.3%) and Ni (7.5%), representing industrial sources. In the rural area, the exact sources of Factor 1 and Factor 3 cannot be determined.

The contributions of these sources to different rice organs were then quantified using a Bayesian mixing model (MixSIAR) with the three PMF factors as source end-members (Figure 4e–h). In all areas, the natural source contributed substantially to leaves, husks, and rice grains. This indicated that natural materials, likely as dust or through irrigation, are directly deposited on aerial tissues or taken up by roots and translocate upward. Industrial and traffic sources showed variable contributions. In the roadside area, the traffic source (Factor 2) contributed 38.5% to leaves and 19.4% to rice grain, while the industrial source (Factor 3) contributed mainly to roots (28.3%). In the industrial area, industrial sources (Factor 1 and Factor 3) contributed 31.9% and 31.9% to roots, respectively, but only 10.7% and 10.0% to leaves, indicating limited impact on above-ground tissues. In the peri-urban area, the mixed pollution source (Factor 2) dominated leaves (18.8%) and husks (8.0%), while the industrial source (Factor 3) contributed more to roots (31.2%). In the rural area, the natural source (Factor 1) contributed 19.5% to leaves and 20.4% to rice grains. Specifically, the Bayesian mixing model revealed that the natural source contributed the highest proportion to rice grains (48.3–70.6%) across all four source areas. Except for natural sources, industrial sources dominated in industrial areas (29.1%), traffic emissions prevailed in roadside areas (19.4%), while mixed sources contributed the most to rice grain contamination in peri-urban areas (28.4%). The peri-urban and rural areas exhibited mixed patterns, reflecting the combined influence of industrial, traffic, and agricultural activities.

### 2.5. Human Health Risk Assessment of PTE Accumulation to Local Residents

**Figure 5:**
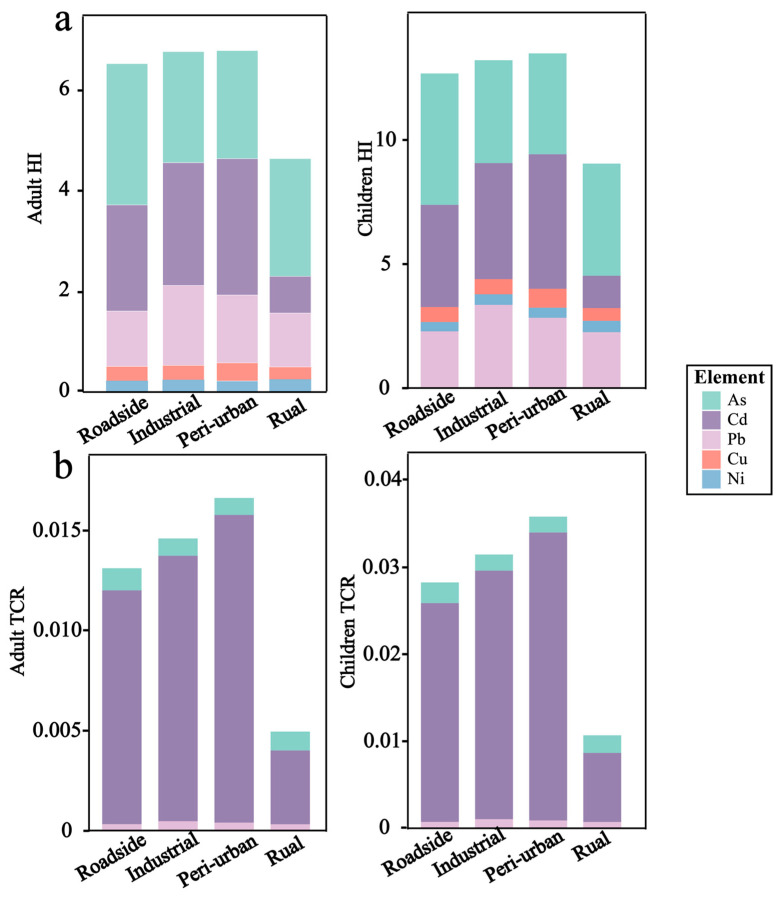
Health risks associated with rice consumption, soil ingestion, and skin contact for adults and children in different source regions (**a**); carcinogenic risks for adults and children in different source regions (**b**).

The health risks posed by PTE contamination to residents were evaluated for both adults and children based on As, Cd, Cu, Ni, and Pb contents in soil and rice grains (Figure 5a). For adults, the health risks varied across source areas, with the peri-urban area showing the highest total hazard index of 6.80, followed by the industrial area (6.78), the roadside area (6.53), and the rural area (4.63) (Figure 5a). Rice ingestion remained the dominant pathway across all areas, accounting for over 90% of the total risk (Figure S3). For rice ingestion, hazard quotients of As ranged from 2.15 to 2.81, Cd from 0.73 to 2.71, and Pb from 1.07 to 1.59 across areas. For children, health risks were substantially higher than for adults. The HI varied across source areas: peri-urban (13.52) > industrial (13.25) > roadside (12.71) > rural (9.07). Element-specific analysis revealed that As, Cd, and Pb were the primary contributors. Soil ingestion risks were mainly from As, while Cd dominated dermal exposure. Carcinogenic risk (CR) was assessed for As, Cd, and Pb (Figure 5b). For adults, the total carcinogenic risk (TCR) across all areas ranged from 4.96 × 10^−3^ to 1.66 × 10^−2^, all exceeding the USEPA acceptable threshold of 10^−4^, indicating a high carcinogenic potential. For children, TCR values were even higher, ranging from 1.07 × 10^−2^ to 3.58 × 10^−2^. Among the three elements, Cd contributed the most to TCR (70–85%), followed by As and Pb.

## 3. Discussion

### 3.1. PTE Pollution in Paddy Fields

The accumulation of PTEs in soil, atmospheric deposition, and various tissues of rice from four anthropogenic source areas (industrial, roadside, peri-urban, and rural) exhibits distinctly different characteristics. A comparison of the studies by Zeng et al. and Li et al. on PTE monitoring data from farmland in Hunan Province indicates that PTE concentrations in soil have declined in recent years [20,21]. In this study, the highest concentrations of Cd, Pb, As, and Ni were found in the soil of the industrial area, with Cd being the primary contaminant. This finding is consistent with previous studies in the Xiangjiang River basin of Hunan Province, where elevated Cd and Cr concentrations in rice grains have been attributed to a high background of soil PTEs and abundant mineral resources in the region [22]. A survey covering farmland in Hunan Province showed that, among the average concentrations of Cd, Pb, Cr, Hg, and As in topsoil, only Cd (0.42 mg/kg) exceeded soil risk in China screening value (0.3 mg/kg in GB15618-2018), and the Pb and Cd in topsoil primarily originated from industrial and transportation activities [23]. In contrast, atmospheric deposition also displayed pronounced spatial variation. The roadside area recorded the highest deposition fluxes for As, Cd, Ni and Pb. A study across three functional areas in Hunan reported that the highest concentrations of As, Ni, Cd, and Pb in atmospheric deposition were also found along the roadside [7]. In industrial areas, atmospheric deposition is considered a major source of Cd. In the industrial and mining areas of Zhuzhou, atmospheric deposition accounts for 96.42% of total Cd inputs [24]. In peri-urban areas, the combined effects of industrial proximity and peri-urban characteristics likely account for the moderate sedimentation flux observed in this region.

Overall, in rice grains, the levels of Cd and Pb in both industrial areas and peri-urban areas exceeded the Chinese food safety standard (0.2 mg/kg in GB 2762-2022). A province-wide survey conducted in 14 prefecture-level cities in Hunan Province showed that the average concentrations of Cd and Pb in rice were 0.28 mg/kg and 0.66 mg/kg, respectively [25]. These values are lower than those observed in the peri-urban (Cd 0.44 mg/kg) and industrial (Cd 0.38 mg/kg, Pb 0.94 mg/kg) areas in this study, but higher than those in rural areas (Cd 0.10 mg/kg), reflecting the severity of the impact of industrial and mixed pollution sources within the study area.

It is noteworthy that the peri-urban area had lower soil Cd (0.70 mg/kg) than the industrial area (0.88 mg/kg), yet its rice grain Cd (0.44 mg/kg) was the highest among all regions. This counterintuitive pattern suggests that mixed pollution sources (industrial, traffic, domestic) may supply Cd in more bioavailable forms, higher exchangeable fractions or that irrigation water with elevated Cd contributes directly to grain accumulation. The soil physicochemical properties were not measured in this study, and we recommend that future studies include these parameters to better understand PTE bioavailability and translocation mechanisms.

### 3.2. Source Apportionment, Pathway Validation, and Implications for Risk Management

Accurate source apportionment of PTEs in paddy fields is a prerequisite for developing targeted pollution control strategies. The PMF can effectively resolve source fingerprints based on elemental covariance. However, it remains subject to interpretational ambiguity arising from overlapping source profiles and lacks a rigorous uncertainty quantification for source contributions. By contrast, the Bayesian MixSIAR model provides a probabilistic framework that incorporates multiple sources of uncertainty and delivers posterior distributions of source proportions. Consequently, the integration of PMF with MixSIAR in this study establishes a more robust and objective methodology for tracing PTE origins and allocating source contributions to specific rice organs. The PMF model analysis identified three source categories across the four regions: natural sources, industrial sources, and traffic sources, each with region-specific elemental fingerprints and contribution ratios. In all four regions, natural sources accounted for 48.3–70.6% of the contributions to rice grains, reflecting Hunan Province’s high geochemical background, as well as factors such as irrigation water and background atmospheric deposition. This finding is consistent with the results of Wang et al. (2018), which indicate that PTE contamination in soils is partly attributable to natural sources [25]. A PMF study of farmland also found that Pb and Cd primarily originate from industrial and traffic activities, while As and Cr primarily originate from soil parent material, and Hg primarily originates from atmospheric deposition [26]. In this study, the proportion attributable to industrial sources was consistent with the PMF results obtained for an industrial center; industrial activities in that region contributed 24.9% to the accumulation of PTEs [27]. In peri-urban areas, mixed sources account for 28.4% of particulate matter, reflecting the combined impact of industry, traffic, and agricultural inputs. A study by Xiao et al. (2023) in Dongting Lake, Hunan, also indicated that agricultural activities accounted for 36.98% and natural sources for 32.94% [28]. Based on their survey of rural areas in Xiangtan County, industrial activities, agricultural activities, and natural sources all have a significant impact on PTE concentrations in farmland soil [29]. A study conducted in Chenzhou, Hunan, identified the primary sources of PTEs in soil, in descending order: ferrous PTEs from mining and smelting (29.1%), atmospheric deposition (22.1%), non-ferrous PTEs from mining and processing (20.7%), combined industrial and transportation sources (14.7%), and natural sources (13.5%) [30]. It is important to note that the contribution of natural sources in rice grains in this study (48.3–70.6%) was higher than that reported in the literature for natural sources in soil (17.4–30.1%) [31]. This discrepancy is not contradictory but reflects the different media and models: soil-only PMF analyses typically classify only geogenic parent material as the natural source, whereas the MixSIAR model additionally incorporates natural inputs such as atmospheric deposition, and the intrinsic capacity of rice plants to take up PTEs from the soil parent material [32]. Moreover, the geo-accumulation index for Cd in the rural area (Igeo = 1.53) already indicates slight enrichment above the natural background, suggesting that even clean areas are affected by long-range atmospheric transport and diffuse pollution [33]. The combined PMF-MixSIAR model not only identifies sources but also quantifies their contributions to specific rice parts, thereby distinguishing between foliar absorption and root uptake pathways [34]. The findings challenge the traditional assumption that “root uptake is the primary route of absorption for all elements.” For example, the traffic source contributed 38.5% to leaves but only 19.4% to grains and 28.3% to roots, demonstrating that PTEs in the atmosphere enter the grain mainly via foliar uptake and subsequent phloem transport, a pathway that bypasses the root barrier [35,36]. This suggests that the use of foliar inhibitors should be considered for the prevention and control of PTE contamination in rice in roadside areas.

In addition, the health risk assessment showed that the risk index is highest in the peri-urban area (6.80) > the industrial area (6.78) > the roadside area (6.53) > the rural area (4.63). Therefore, in risk management, peri-urban areas require special attention, as the interaction of multiple pollution sources exacerbates the contamination burden on rice grains. In contrast, the rural area showed the lowest health risk. In addition, as the most economically valuable staple crop, rice accounts for more than 90% of the total risk. It is worth noting that As, Cd, and Pb are priority elements that require urgent measures to reduce emissions. Although the As content in rice grains from various regions is not particularly high, it still poses a significant risk to human health due to differences in the Reference Intake (RfD) for different elements. These findings are consistent with Tang et al. (2019) and Yu et al. (2023) [4,37].

### 3.3. PTE Translocation in the Atmospheric Deposition–Soil–Rice System

Rice accumulates PTEs through two main pathways: root uptake from the soil and foliar uptake from the atmosphere. In industrial areas, elevated Cd concentration in roots (5.67 mg/kg) and rice grain (0.38 mg/kg) confirmed soil-to-root transfer as the dominant pathway [38]. Source apportionment showed that industrial sources contributed 31.9% to roots, indicating that industrial emissions mainly affect rice through root uptake [39,40,41]. In roadside areas, by contrast, leaves accumulated the highest contents of Pb (1.40 mg/kg) and As (2.99 mg/kg), matching the high atmospheric deposition and supporting foliar uptake as a major pathway [42]. Xia et al. (2023) reported that rice leaves can directly absorb Cd, accounting for 52–70% of the total accumulation in rice grains, exceeding the contribution of root uptake (30–48%) [13]. Zhou et al. (2024) also indicated that rice leaves could absorb newly deposited Cd, accounting for 45–49% of the total accumulation in rice grains [12]. Notably, the BCF of atmosphere-to-leaf for Cd reached 6.61 in roadside areas, suggesting that Cd from traffic sources in atmospheric deposition may be present in a form that is particularly easily absorbed by plant leaves. Studies have shown that 30–84% of Cd in dust is present in water-soluble and exchangeable fractions, while mobile Pb is mainly associated with carbonates and reducible phases in Asia [43]. In the roadside area, the traffic source contributed 38.5% to leaves and 19.4% to rice grains. Meanwhile, the industrial source contributed mainly to roots (28.3%), confirming that traffic-derived PTEs enter the plant primarily via atmospheric deposition onto leaves. Previous studies have also shown that 75–90% of atmospheric PTEs are adsorbed onto particles or aerosols, which can then deposit on crop surfaces and be absorbed by plants [44]. Atmospheric PTEs generally exhibit higher bioavailability than those in surface soils [45,46,47]. It is worth noting that, although soil and atmospheric deposition levels in peri-urban areas were moderate among the four regions, Cd (0.44 mg/kg) levels in rice were the highest, and Pb (0.80 mg/kg) levels were also relatively high. The peri-urban area exhibits mixed-source characteristics, with mixed pollution sources accounting for 28.4% of rice grain contamination, while industrial pollution sources account for 23.2%. This indicates the combined impact of pollution sources from industry, transportation, residential activities, and agricultural activities [48,49,50]. In this study, the PMF and MixSIAR models, combined with PTE concentrations in different rice parts and the corresponding bioconcentration and transfer factors, were used to elucidate the accumulation pathways of PTEs in rice grains across different regions. This mixed source profile likely explains why the peri-urban area, despite having no single dominant source, had the highest total health risk (HI 6.80). In contrast, the rural area showed the lowest PTE concentrations in soil, air, and rice tissues, and the lowest health risk. In the rural area, the natural source still dominated grains (56.0%), but industrial and unidentified sources contributed 20.4% and 23.6%, respectively, indicating long-range atmospheric transport of industrial pollutants [51,52]. The findings indicate that foliar absorption is the more efficient pathway for Cd under high atmospheric deposition. This has significant implications for transfer through the food chain: rice leaf tissue with elevated Cd levels may be ingested directly by livestock, thereby introducing cadmium into the animal-derived food chain. Rice husks also need attention because it is an intermediate storage organ between leaves and grains, receiving PTEs from both upward translocation and direct atmospheric deposition, and because husk is commonly used as animal feed in rural areas, potentially introducing PTEs into the food chain [53,54]. This pathway has received little attention to date.

## 4. Materials and Methods

### 4.1. Study Area Description

This study focuses on six key cities within Hunan (108°47′–114°15′ E, 24°38′–30°08′ N): Changsha, Zhuzhou, Xiangtan, Liuyang, Liling, and Ningxiang. This selection encompasses the core areas of urbanization, economic activity, industrial manufacturing, and agricultural production within the province. The measured soil pH in our study ranged from 5.36 to 6.14, and soil organic matter (SOM) ranged from 18.9 to 30.0 g/kg. The soil types were predominantly red earth paddy soil and yellow earth paddy soil, which are the two dominant soil groups in Hunan Province. Roadside (*n* = 26) represents sampling sites located within 1 km of highways; industrial (*n* = 23) represents sampling sites located within 2 km of industrial zones; peri-urban (*n* = 20) represents sampling sites located in the urban–rural transition; rural (*n* = 20) represents sampling sites located more than 10 km from the pollution sources, where over 80% of the land is farmland. See Supplementary File S1.1 Study area for details.

### 4.2. Sample Collection and Analysis

Field sampling was conducted from July to September 2024 across 99 selected sites within the study region. At each site, triplicate paddy soil samples were collected alongside in situ atmospheric dry–wet deposition and mature rice plants, forming a matched sample set. The geographic coordinates of all sampling points were recorded using a portable GPS unit. To ensure representativeness and prevent contamination, composite soil samples (≥1 kg per site) were obtained by homogenizing sub-samples collected with non-metallic tools, carefully avoiding obviously disturbed areas such as field ridges and ditches. The samples were sealed, transported to the laboratory, air dried, ground, and sieved through 20-mesh and 100-mesh nylon sieves for storage and subsequent analysis.

Atmospheric deposition samples were collected using dust collection cylinders, which were cylindrical jars (plastic) with an inner diameter of 20 cm and a height of 60 cm. The cleaned dust collection cylinders underwent a rigorous preparation process, involving immersion in 10% (*v*/*v*) HCl for a duration of 24 h. The collectors were thoroughly rinsed with 500 mL of deionized water, and the total was transferred to 1 L PET bottles. Subsequently, these cylinders were positioned on the roof of a residential house adjacent to the sampling fields. To ensure comprehensive data collection, the dust collection cylinders were replaced every three months throughout the entire rice-growing season. Concurrently, soil samples were extracted from the tillage layer (0–15 cm) within the two study areas and transported to the laboratory for air drying using plastic bags. Upon the maturation of the rice, three plants exhibiting relatively uniform growth were selectively chosen from each plot. The rice plants were soaked in 20 mM EDTA-2Na solution for 30 min and then carefully rinsed with ultrapure water. Rice samples were taken back to the laboratory, processed, dried and finally divided into roots, stem base, leaves, rachis, husk and grain.

Soil samples were air dried at room temperature, crushed and passed through a 0.145 mm sieve before determination of PTE concentrations. The grain, shoot, and root of rice samples were firstly deactivated by oven drying at 105 °C for 30 min, dried in an oven at 65 °C until they reached constant weight, and then crushed with a pulverizer. Soil samples were digested with HNO_3_-H_2_O_2_-HF (6:3:3, HNO_3_ 10 mL), and plant samples were digested with HNO_3_-H_2_O_2_ (2:1, HNO_3_ 10 mL). The HNO_3_ used is 65% (*v*/*v*) and of guaranteed reagent. Filtration separated the atmospheric deposition samples into dry and aqueous ones. Dry samples were air dried and then digested in the same way as the soils. The aqueous samples were extracted at 50 mL and acidified by hydrochloric acid, then digested with 2 mL HNO_3_ and 1 mL HCl at 85 °C until the samples evaporated to 20 mL. Each batch of analysis included sample replicates and reagent blanks to ensure the analysis’s quality. All samples were stored in the dark at 4 °C in a refrigerator and measured within 1 month. Certified reference materials (CRMs), including soil CRM (GBW07457, GSS-28) and rice CRM (GBW10045a, GSB-30), both from the National Research Center for Certified Reference Materials (Beijing, China), were used to validate the digestion and analytical procedures. Each batch of analysis included sample replicates and reagent blanks to ensure the analysis’s quality. All samples were stored in the dark at 4 °C in a refrigerator and measured within 1 month. The concentrations of PTEs in the solution were determined by inductively coupled plasma mass spectrometry (ICP-MS, Nexion 350X, PerkinElmer, USA), the limit of detection was 0.01 μg/mL for all elements, and recovery rates were between 90 and 110%.

### 4.3. Soil Pollution Assessment

The assessment considered three primary exposure pathways: rice grain consumption, soil ingestion, and dermal contact with soil particles. The risk to human health from PTEs in soil is mainly due to long-term ingestion and dermal contact with contaminated soil particles. The risk to human health from PTEs in rice grain is mainly due to the consumption of contaminated rice.

(1) $$P_{n}=\sqrt{\left(P_{imax}^{2}+{\bar{P}}^{2}\right)/2}$$

(2) $$P_{i}=C_{i}/S_{i}$$

(3) $$\bar{P}=\frac{1}{n}\sum_{i=1}^{n}P_{i}$$

Soil PTE contamination in the study area was evaluated using the Nemerow Integrated Pollution Index (P_n_) where P_i_ is the pollution index of each element, i represents the different PTEs, C_i_ represents the concentration of PTE elements in the soil, and S_i_ represents the mass standard values of different PTEs. In this study, the secondary standard value in China’s soil environmental quality standard (GB15618-2008) was used as the standard value. P_imax_ is the maximum pollution index of all elements, and $\bar{P}$ is the average value of the pollution index.

### 4.4. Bioconcentration and Transfer Coefficients

The bioconcentration factors (*BCF_s_* and *BCF_a_*) and transfer factor (*TF*) were calculated as detailed in the Supplementary Materials. *BCF_s_* and *BCF_a_* represent the bioconcentration factors from soil and atmospheric deposition, respectively. *C_a_* represents the concentration of PTEs in atmospheric deposition (μg/m^2^/day). *C_S_* represents the concentration of PTEs in soil (mg/kg), where C_ul_ (mg/kg) represents the concentration in each part of the plant, and *C_l_* (mg/kg) represents the PTE concentration in the lower part of the rice system compared to the upper part.

(4) $${\mathrm{BCF}}_{S}=\frac{C_{ul}}{C_{S}}$$

(5) $${\mathrm{BCF}}_{a}=\frac{C_{ul}}{C_{a}}$$

(6) $$\mathrm{TF}=\frac{C_{ul}}{C_{L}}$$

### 4.5. Health Risk Assessment


(7) $${ADI}_{rice}=\frac{C_{grain}\times IR\times EF\times ED}{AT\times BW}\times 10^{-6}$$

(8) $${ADI}_{ing}=C_{soil}\times \frac{IngR\times EF\times ED}{BW\times AT}\times 10^{-6}$$

(9) $${ADI}_{dermal}=C_{soil}\times \frac{SA\times AF\times ABS\times EF\times ED}{BW\times AT}\times 10^{-6}$$

(10) $$HI=\sum {HQ}_{ij}=\sum \frac{{ADI}_{ij}}{{RfD}_{i}}$$

(11) CR=ADI×SF

(12) $$\mathrm{TCR}=\sum {CR}_{ij}$$

The potential non-carcinogenic health risks and carcinogenic health risks to adults and children (2–6 years) from PTEs were evaluated following the methodology established by the U.S. Environmental Protection Agency (USEPA) [55,56]. The non-carcinogenic health risks assessment considered three primary exposure pathways: rice grain consumption, soil ingestion, and dermal contact with soil particles. The risk to human health from PTEs in soil is mainly due to long-term ingestion and dermal contact with contaminated soil particles. The risk to human health from PTEs in rice grain is mainly due to the consumption of contaminated rice. ADI_rice_, ADI_ing_, and ADI_dermal_ represent the average daily intake (mg/kg/day) via rice consumption, soil ingestion, and dermal contact with soil, respectively; C_soil_ is the concentration of PTEs in soil (mg/kg); C_grain_ is that in rice grain (mg/kg); IR is the rice ingestion rate (mg/person/day); IngR is the soil ingestion rate (mg/day); EF is exposure frequency (days/year); ED is exposure duration (years); BW is body weight (kg); AT is averaging time (AT = 365 × ED, days); SA is exposed skin surface area (cm^2^); AF is adherence factor (kg/cm^2^/day); and ABS is the dermal absorption factor. The corresponding RfD values for each element are provided in the Supplementary Materials (Table S1).

### 4.6. Source Apportionment and Bayesian Mixing Model

To identify potential sources of PTEs in the atmospheric–soil–rice system, non-negative matrix factorization was performed using the NMF package in R (version 4.1.0). The input data matrix consisted of concentrations of As, Cd, Cr, Cu, Ni, Pb, and Zn in all samples, including soil, atmospheric deposition, and rice organs (root, leaf, husk, and rice grain). The factorization was run with ranks from 2 to 6, and the optimal number of factors was determined based on the cophenetic correlation coefficient and residual sum of squares. Three factors were finally retained. Each factor was interpreted by examining its elemental loadings, and factor contributions to individual samples were extracted for subsequent analyses.

To quantitatively apportion the contributions of the identified sources to PTEs in different rice organs, a Bayesian mixing model was implemented using the MixSIAR package in R. The model considered the three PMF-derived factors as source end-members for each area. The mean and standard deviation of each element within each factor were calculated from the factor loading matrices. The model was run separately for each rice organ (root, leaf, husk, rice grain) with 3 Markov chain Monte Carlo chains, each consisting of 300,000 iterations, with a burn-in of 200,000 and thinning by 100. Convergence was assessed using the Gelman–Rubin diagnostic (all < 1.05). The posterior distributions of source proportions were summarized as mean and standard deviation.

### 4.7. Atmospheric Deposition Flux

Generally, atmospheric deposition flux is used to represent the intensity of atmospheric deposition, which refers to the mass of dry and wet atmospheric deposition per unit area per unit time. The calculation formula for atmospheric deposition flux is:

(13) $$Q=\frac{M}{S\times d}$$

(14) $$M=M_{\mathrm{dry}}+M_{\mathrm{wet}}$$

(15) $$M_{\mathrm{wet}}=C\;\times \;V_{\mathrm{dig}}\;\times \;\frac{V_{\mathrm{total}}}{V_{\mathrm{aliquot}}}$$

where Q is the deposition flux of PTEs (μg/m^2^/day); S is the area of the dust collector (m^2^); M is the total mass of deposition of PTEs (μg); and d is the number of sampling days. C is the measured concentration in the digested solution (μg/mL); V_dig_ is the final constant volume of the digestion solution (mL); V_total_ is the total volume of the wet deposition sample (mL); and V_aliquot_ is the aliquot volume taken from the sample for digestion (mL). The ratio V_total_/V_aliquot_ is the aliquot factor.

### 4.8. The Geo-Accumulation Index

To better distinguish between natural and anthropogenic contamination sources, we calculated the geo-accumulation index (Igeo) for each area, using the China National Environmental Monitoring Centre (1990). The geo-accumulation index is a widely used quantitative measure of the degree of anthropogenic enrichment, defined as:

(16) $$\mathrm{Igeo}\;=\;\log_{2}(\frac{\mathrm{Ci}}{1.5\times \mathrm{Bi}})$$

where Ci is the measured concentration of element i in soil, and Bi is the corresponding geochemical background value for Hunan Province. The geo-accumulation indices of various elements in different functional zones are in Table S3.

### 4.9. Data Analysis

Data analysis was performed using Microsoft Excel 2019, and the corresponding charts were created in R Studio 4.5.2 using packages such as ggplot2, plyr, ggthemes, NMF, and MixSIAR, etc.

## 5. Conclusions

The study indicates significant variations in PTE contamination characteristics, rice uptake pathways, source apportionment, and health risk profiles among different source areas. The concentrations of Cd and Pb were highest in soil in the industrial area. The roadside area had the highest atmospheric concentrations of Pb and As. Consequently, root contamination was the most severe in the industrial area, while leaf contamination was the most severe in the roadside area. The peri-urban area showed the highest Cd content in rice grains (0.44 mg/kg) and overall health risk (HI 6.80). In contrast, the rural area was relatively clean. In particular, the accumulation of As, Cd, and Pb in rice grains needs to be of concern. The health risk assessment revealed that rice ingestion accounts for more than 90% of the total non-carcinogenic and carcinogenic risks for both adults and children, with peri-urban and industrial areas posing the highest risks. Therefore, consumption of rice grains in these areas may pose a significant health risk, particularly for children. Moreover, the results of the study suggest that the effect of atmospheric deposition on PTE accumulation in paddy fields should receive more attention. The contribution of atmospheric deposition to PTE contamination of rice grains should not be underestimated, especially for Cd. Therefore, this study recommends region-specific mitigation strategies. This study can provide data to support the government and related organizations to prevent and control PTE pollution and contribute to ensuring food safety.

## Figures and Tables

**Table 1:** Bioconcentration and transport factors of PTEs from different sources in rice plants.

Area		As	Cd	Ni	Pb	Cr	Cu	Zn
**The Industrial Area**	*BCF* *_soil-to-root_*	3.81	5.25	0.75	0.64	2.57	0.75	0.52
	*BCF* *_atmosphere-to-leaf_*	2.55	0.42	0.11	0.07	1.74	0.14	0.47
	*TF* *_root-to-leaf_*	0.04	0.45	0.06	0.05	0.34	0.31	1.30
	*TF* *_leaf-to-husk_*	0.18	1.48	1.92	0.39	4.95	0.68	0.33
	*TF* *_root-to-husk_*	0.005	0.08	0.04	0.02	0.10	0.09	0.31
	*TF* *_leaf-to-grain_*	0.04	0.39	0.44	1.14	0.88	0.33	0.25
	*TF* *_root-to-grain_*	0.001	0.13	0.03	0.03	0.03	0.08	0.32
	*TF* *_husk-to-grain_*	0.29	2.00	0.62	3.16	0.24	0.69	1.52
**The Roadside Area**	*BCF* *_soil-to-root_*	2.79	4.24	0.70	0.48	4.60	0.75	0.46
	*BCF* *_atmosphere-to-leaf_*	1.15	6.61	0.61	0.13	0.75	0.43	0.46
	*TF* *_root-to-leaf_*	0.04	0.30	0.08	0.06	0.09	0.24	1.37
	*TF* *_leaf-to-husk_*	0.25	0.27	1.44	0.41	6.20	0.52	0.29
	*TF* *_root-to-husk_*	0.005	0.05	0.06	0.02	0.20	0.15	0.32
	*TF* *_leaf-to-grain_*	0.04	0.30	0.72	1.84	0.69	0.37	0.33
	*TF* *_root-to-grain_*	0.001	0.07	0.03	0.03	0.05	0.10	0.35
	*TF* *_husk-to-grain_*	0.24	1.88	0.86	2.30	0.23	0.74	1.99
**The Peri-Urban Area**	*BCF* *_soil-to-root_*	3.60	6.09	0.60	0.59	4.81	0.85	0.63
	*BCF* *_atmosphere-to-leaf_*	0.21	0.77	0.08	0.04	2.18	0.22	0.76
	*TF* *_root-to-leaf_*	0.05	0.20	0.07	0.05	0.18	0.29	0.93
	*TF* *_leaf-to-husk_*	0.24	0.78	1.83	0.40	8.53	0.81	0.31
	*TF_root-to-husk_*	0.005	0.06	0.07	0.02	0.17	0.13	0.28
	*TF* *_leaf-to-grain_*	0.04	0.38	0.75	1.65	0.71	0.39	0.32
	*TF* *_root-to-grain_*	0.001	0.08	0.05	0.03	0.04	0.10	0.27
	*TF* *_husk-to-grain_*	0.21	1.52	1.03	2.58	0.19	0.83	1.84
**The Rural Area**	*BCF* *_soil-to-root_*	3.65	4.47	0.66	0.65	3.83	0.76	0.44
	*BCF* *_atmosphere-to-leaf_*	0.35	0.43	0.13	0.03	0.84	0.06	0.48
	*TF* *_root-to-leaf_*	0.04	0.30	0.05	0.06	0.13	0.24	1.21
	*TF* *_leaf-to-husk_*	0.23	0.80	2.70	0.46	6.30	0.82	0.35
	*TF* *_root-to-husk_*	0.005	0.11	0.06	0.02	0.15	0.15	0.35
	*TF* *_leaf-to-grain_*	0.05	0.26	1.25	1.15	0.63	0.37	0.33

## Data Availability

The raw data generated for this study are available upon request to the corresponding author.

## Supplementary Materials

The following supporting information can be downloaded at: https://www.mdpi.com/article/10.3390/plants15121818/s1. 1.1 Study Area; Table S1: The value of parameters for the calculation of health risk; Table S2: Background values for different soil types; Table S3: The geo-accumulation indices of various elements in different functional; Table S4: Results of ANOVA for heavy metals in soil, atmospheric deposition, and rice plants (F-values and *p*-values); Table S5: The pH and SOM values for different soil types; Figure S1: Map of sampling sites in the study area; Figure S2: Health risks of rice ingestion, soil ingestion, and dermal contact under different source areas; Figure S3. Monte Carlo simulation analysis of health risks and carcinogenic risks in adults and children.
