# Supplementary material for: Source-Specific Accumulation, Translocation, and Health Risks of Potentially Toxic Elements in Paddy Fields from Different Anthropogenic Impact Zones in Hunan Province, China
Source: Plants (Basel). 2026 Jun 12;15(12):1818. doi: 10.3390/plants15121818 (PMC13306372; doi:10.3390/plants15121818)
Supplement: Supplementary file 1 [file plants-15-01818-s001.zip › plants-4303205-supplementary.pdf]

Supplementary Material

S1. **Manuscript title**: Source-Specific Accumulation, Translocation, and Health Risks of Heavy Metals in Paddy Fields from Different Anthropogenic Impact Zones in Hunan Province, China

S2. Number of pages: 10

S3. Number of table: 4

S4. Number of figure: 3

S1.1 Study Area

This study focuses on six key cities/counties within Hunan Province (108°47′–114°15′ E, 24°38′–30°08′ N): Changsha, Zhuzhou, Xiangtan, Liuyang, Liling, and Ningxiang. This selection encompasses the core areas of urbanization, economic activity, industrial manufacturing, and agricultural production within the province. According to the 2023 Hunan Statistical Yearbook:

Changsha County (under Changsha's administration) has a permanent resident population of 1.4347 million. Its GDP reached 212.95 billion RMB in 2023. The county's grain planting area was 112.05 thousand hectares, with a total grain output of 495,000 tons. Changsha County is a major industrial hub in Hunan Province, known for its advanced manufacturing sectors including engineering machinery, automotive and parts, hosting 562 industrial enterprises above designated size.

Zhuzhou has a population of 3.85 million and a secondary industry value of 161.39 billion RMB, driven by non-ferrous metal smelting, rail transit equipment, and aerospace manufacturing.

Xiangtan has a population of 2.70 million and a secondary industry value of 138.15 billion RMB, with steel, mining machinery, and automotive manufacturing as pillar industries.

Liling has a permanent resident population of 0.87 million and a GDP of 88.97 billion RMB (2023). It is a major fireworks production base with 176 licensed enterprises (annual output >20 billion RMB), and also has a long history of non-ferrous metal mining and processing (Pb, Zn, Cd, As), with scattered small‑scale mines and smelters contributing to regional heavy metal emissions.

Liuyang and Ningxiang (county-level cities under Changsha’s administration) are major grain-producing areas and industrial hubs. Liuyang is known for fireworks and biomedicine, Liling for ceramics and building materials, and Ningxiang for food processing and garment manufacturing.

The four anthropogenic source areas were defined based on the following criteria:

Roadside area: Sampling sites located within 1 km of major highways or urban roads in the ChangZhuTan region. According to the 2023 Hunan Transport Statistical Bulletin, the national highway network in Hunan carries an average annual daily traffic (AADT) of 18,977 vehicles (standard passenger car equivalent), while the ChangZhuTan expressway network records an AADT of 32,616 vehicles. The high density of vehicle movement results in continuous emissions of Pb, As, and other traffic-related pollutants.

Industrial area: Sampling sites located within 2 km of active industrial zones, including non-ferrous metal smelters, electroplating plants, machinery, and petrochemical facilities. Zhuzhou alone hosts over 500 industrial enterprises above designated size, including several large-scale non-ferrous metal processing plants, generating substantial industrial emissions.

Peri-urban area: Sampling sites in the transition zones between urban centers and rural farmlands, characterized by mixed land use. These areas receive combined inputs from industrial, traffic, and residential/agricultural sources, with Changsha's suburban counties having a resident population density of approximately 3,500 persons/km^2 and industrial output accounting for nearly 30% of local GDP.

Rural area: Sampling sites located >10 km away from major industrial zones and highways, with agricultural land use >80% and population density <300 persons/km^2. These areas were designated as background reference sites, though some long-range atmospheric transport may still occur.

Table S1. The value of parameters for the calculation of health risk

	**Cu**	**Ni**	**As**	**Cd**	**Pb**	**References**
RfDing	0.04	0.02	0.0003	0.001	0.0035	USEPA 2015
RfDdermal	0.012	0.0054	0.000123	0.00001	0.000525	USEPA 2015
ABS	0.1	0.35	0.03	0.14	0.006	HC 2004

	**Value**	**References**		**Value**	**References**
IR	342.9×10^3 (198×10^3)	ZJFDA 2008	BW	55.9 (15)	Ge, 1992
EF	345	Smith, 1994	IngR	100 (200)	USEPA, 1997
ED	70 (6)	USEPA, 1997	SA	5700 (2373)	USEPA, 1997
AF	0.2	USEPA, 1997			

Table S2. Background values for different soil types.

Soil types	As	Cd	Cr	Cu	Ni	Pb	Zn	Ca	Mg
Red earths (mg/kg)	13.6	0.065	62.6	24.4	25.7	29.1	80.1	800	3400
Yellow earths (mg/kg)	12.4	0.080	55.5	21.4	25.3	29.4	79.2	1200	4200

Table S3. The geo-accumulation indices of various elements in different functional zones. I_{geo} <0 practically unpolluted, 0-1 unpolluted to moderately polluted, 1-2 moderately polluted, 2-3 moderately to strongly polluted, 3-4 strongly polluted, 4-5 strongly to very strongly polluted, and >5 very strongly polluted.

	**As**	**Cd**	**Ni**	**Pb**	**Cr**	**Cu**	**Zn**
The Roadside Area	0.59	2.12	-0.67	0.28	-0.28	-0.18	0.03
The Industrial Area	0.29	2.59	-0.60	0.19	-0.15	-0.59	-0.03
The Peri-urban Area	0.19	2.13	-0.96	0.03	-0.55	-0.44	-0.02
The Rural Area	0.04	1.53	-0.89	-0.32	-0.19	-0.54	-0.09

Table S4. Results of ANOVA for heavy metals in soil, atmospheric deposition, and rice plants (F-values and p-values).

Group	Part	Element	F_value	p_value
Soil		As	8.09	< 0.001
		Cd	19.22	< 0.001
		Cr	5.08	0.002
		Cu	12.35	< 0.001
		Ni	9.83	< 0.001
		Pb	20.89	< 0.001
		Zn	1.14	0.332
Atmospheric deposition		As	2.61	0.053
		Cd	3.50	0.017
		Ni	12.68	< 0.001
		Pb	4.63	0.004
		Cr	3.80	0.011
		Cu	3.24	0.023
		Zn	6.15	0.001
Rice Plant	Root	As	0.60	0.615
	Leaf	As	1.75	0.157
	Husk	As	0.21	0.886
	Grain	As	1.87	0.135
	Root	Cd	21.02	< 0.001
	Leaf	Cd	5.43	0.001
	Husk	Cd	3.05	0.029
	Grain	Cd	5.89	0.001
	Root	Cr	1.65	0.178
	Leaf	Cr	2.86	0.037
	Husk	Cr	13.60	< 0.001
	Grain	Cr	3.88	0.010
	Root	Cu	4.11	0.007
	Leaf	Cu	5.37	0.001
	Husk	Cu	8.37	< 0.001
	Grain	Cu	10.22	< 0.001
	Root	Ni	7.83	< 0.001
	Leaf	Ni	6.48	< 0.001
	Husk	Ni	12.67	< 0.001
	Grain	Ni	0.30	0.822
	Root	Pb	12.55	< 0.001
	Leaf	Pb	3.34	0.020
	Husk	Pb	2.34	0.074
	Grain	Pb	3.01	0.031
	Root	Zn	13.50	< 0.001
	Leaf	Zn	2.80	0.040
	Husk	Zn	1.53	0.206
	Grain	Zn	4.13	0.007

Table S5. The pH and SOM values for different soil types.

**Area**	**pH (mean ± SD)**	**pH range**	**SOM (g/kg, mean ± SD)**	**SOM range**
The Roadside Area	5.72 ± 0.12	5.48-5.96	22.3 ± 1.6	19.1-25.5
The Industrial Area	5.67 ± 0.16	5.36-5.98	21.8 ± 1.5	18.9-24.7
The Peri-urban Area	5.75 ± 0.11	5.53-5.97	25.5 ± 2.3	21.0-30.0
The Rural Area	5.83 ± 0.16	5.52-6.14	23.9 ± 1.5	20.9-26.9

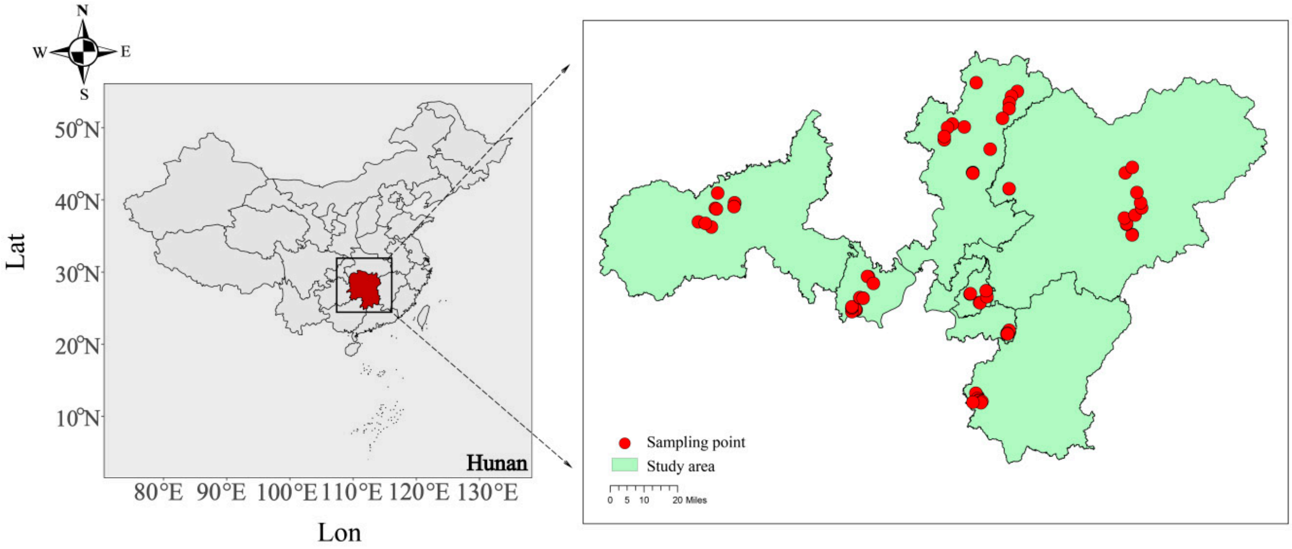

Figure S1. Map of sampling sites in the study area.

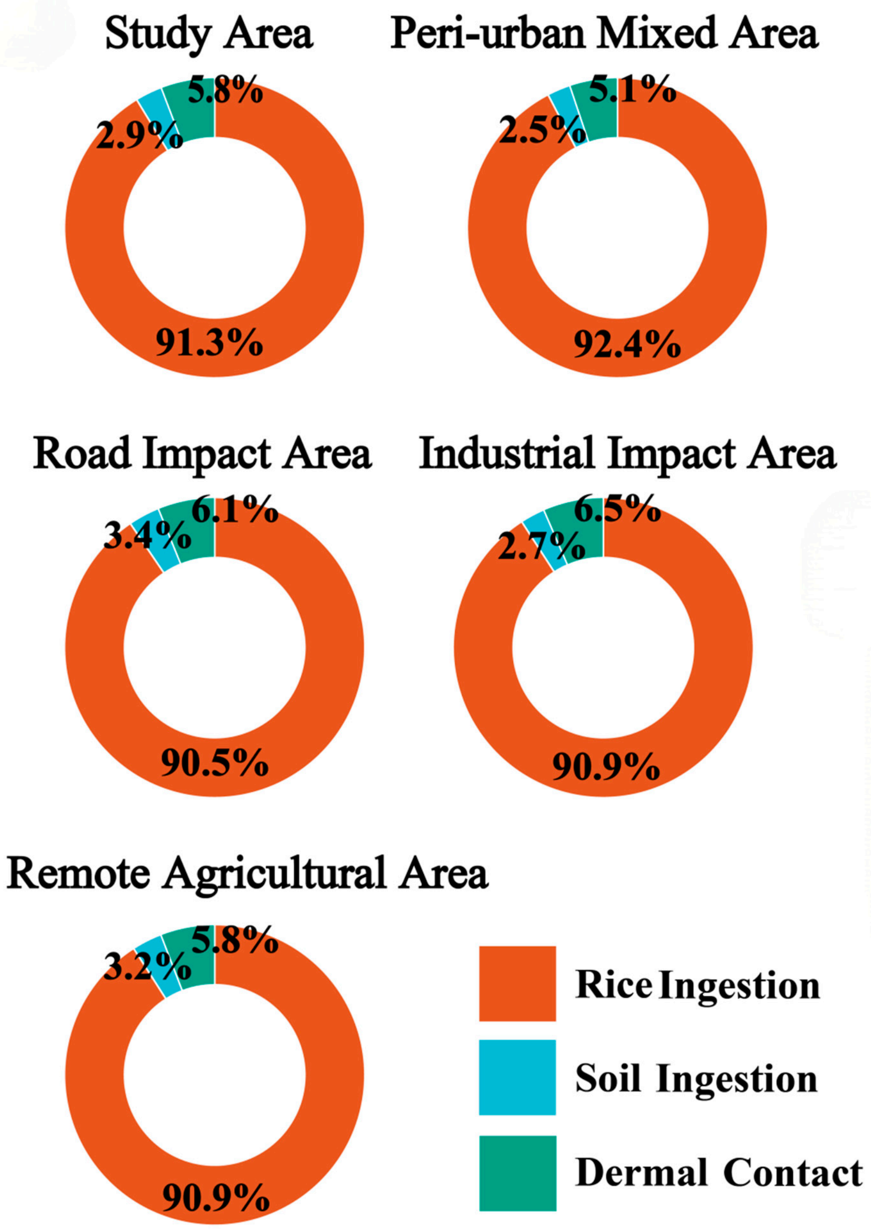

Fig. S2. Health risks of rice ingestion, soil ingestion, and dermal contact under different source areas

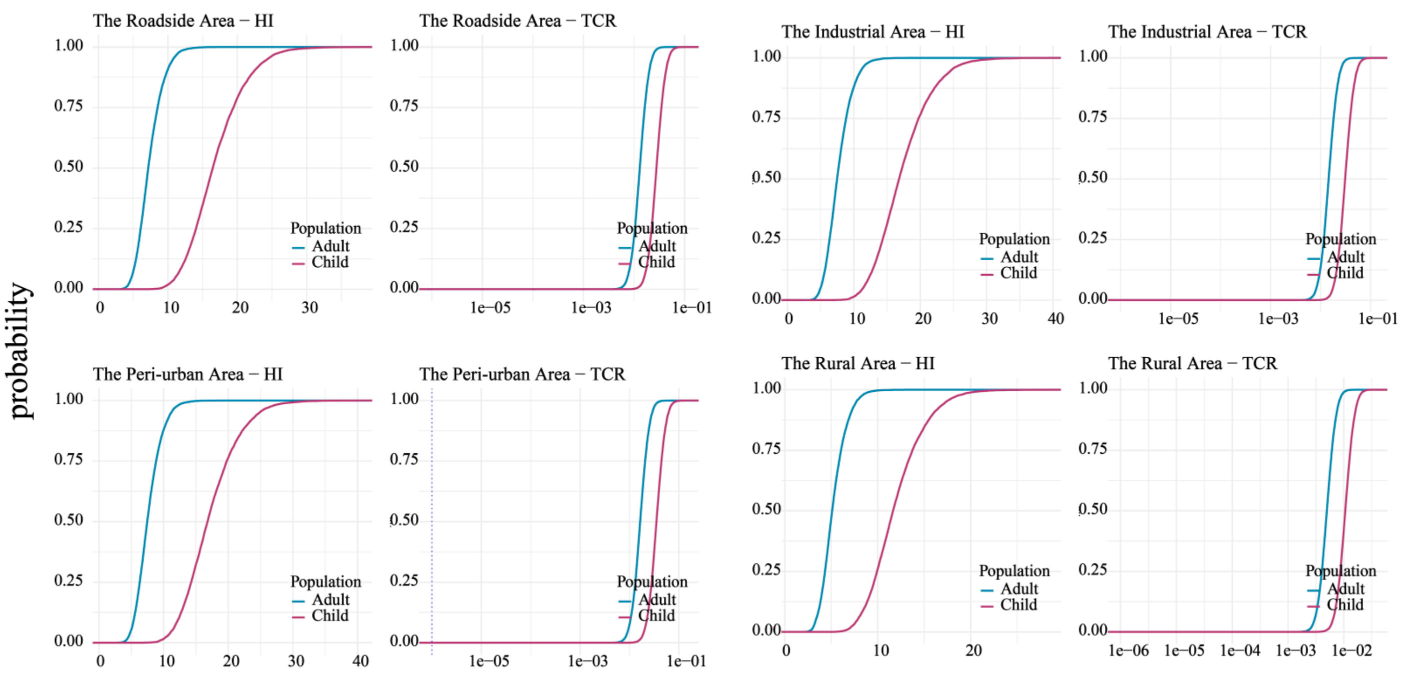

Fig. S3. Monte Carlo simulation analysis of health risks and carcinogenic risks in adults and children.
